# Evaluation of the Risk for Acute Kidney Injury in Adult Cystic Fibrosis Patients Receiving Concomitant Vancomycin and Tobramycin

**DOI:** 10.7759/cureus.1912

**Published:** 2017-12-06

**Authors:** Corinne Muirhead, Jeong Y Lim, Jodi Lapidus, Kelvin MacDonald

**Affiliations:** 1 Doernbecher, Oregon Health and Science University; 2 Knight Cancer Institute, Oregon Health and Science University; 3 Department of Biostatistics, Oregon Health and Science University; 4 Department of Pediatrics, Oregon Health and Science University

**Keywords:** cystic fibrosis, vancomycin, tobramycin, acute kidney injury

## Abstract

Background

The risk for acute kidney injury (AKI) has been associated with both tobramycin and vancomycin.

Objective

To determine whether the rate of drug therapy-related nephrotoxicity is greater in Cystic Fibrosis (CF) patients receiving concomitant vancomycin and tobramycin than patients receiving either agent alone.

Methods

Adult CF patients admitted for acute pulmonary exacerbation (APE) over a seven-year period (2008-2014), who received at least 72 hours of intravenous vancomycin, tobramycin or a combination of the two agents were evaluated for AKI. AKI was defined as a 1.5-fold increase in serum creatinine per RIFLE criteria. One hundred seventy-four hospital encounters from 72 unique patients were assessed in this single-center, cross-sectional study.

Results

AKI outcomes were not statistically different. AKI rates were 19% for vancomycin, 8.7% for tobramycin, and 19.7% for combination cohorts (p = 0.16).

Conclusion

Our data suggest there is no significant difference in AKI risk when vancomycin and tobramycin combination therapy is used.

## Introduction

Cystic Fibrosis (CF) is an autosomal recessive genetic disorder where the Cystic Fibrosis Transmembrane Conductance Regulator (CFTR) protein fails to conduct chloride ions at the apical surface of epithelia. The result is thick retained mucus in the lungs that become permanently colonized with pathogenic bacteria and contribute to lung injury. Ninety percent of CF deaths are the result of pulmonary system disease. An increase in pulmonary symptoms is known as an exacerbation and is treated primarily by antimicrobials to reduce bacterial burden, in addition to airway clearance therapy [1].

Two clinically significant lung bacterial species present in CF mucus are methicillin-resistant *Staphylococcus aureus* (MRSA) and *Pseudomonas aeruginosa *(Pa). Both MRSA and Pa colonization is associated with a more rapid decline in lung function [2]. Overall the prevalence of MRSA in CF sputum cultures has increased 15% over the last 10 years, with a national average of 25.9% in 2014 [2]. Despite a decrease in prevalence, overall, about half of all CF patients are colonized with Pa, and it continues to be the most prevalent bacteria isolated in about 75% of CF patients over 25 years of age [2].

Treatment for CF pulmonary exacerbations is directed by surveillance culture of the respiratory tract for pathogens. The current standard approach for Pa treatment in CF patients is to utilize dual agent antimicrobial coverage for Pa [3]. The most commonly utilized anti-pseudomonal regimen among CF programs includes once daily extended-interval tobramycin and an appropriate anti-pseudomonal beta lactam [4].

The existing CF pulmonary exacerbation care guidelines do not include a discussion of MRSA treatment [3]. The Infectious Diseases Society of America (IDSA) currently recommends vancomycin treatment for MRSA pneumonia in hospitalized patients [5]. Advantages include a low cost of administration, however, therapeutic drug levels are needed and there is a risk of nephrotoxicity [6-10]. Linezolid is also an effective anti-MRSA agent. Linezolid requires laboratory monitoring for myelosuppression and can increase the risk for serotonin syndrome if given with concurrent selective serotonin-receptor inhibitor (SSRI) medications. A 2015 meta-analysis reviewed MRSA treatment in CF patients and concluded that appropriate first-line antimicrobial agents for MRSA treatment include vancomycin and linezolid [11]. A 2015 survey by Zobell, et al. assessed MRSA treatment practices in pediatric and adult CF Foundation-accredited programs. The most commonly reported used inpatient medication was linezolid (34% of pediatric and 35% adult centers), with approximately half of the centers using oral linezolid. Vancomycin is the next most prevalently used (31% pediatric and 30% adult) [12].

Both MRSA and Pa can be found simultaneously in CF cultures. Our center has an overall co-infection rate of 10.5% (pediatric rate of 7% and adult rate of 15.7%); another CF center reported an equivalent combined pediatric and adult rate of 11% [13]. A co-infection rate in our adult CF patients that is double to that of our pediatric patients is not unexpected as older patients with more disease burden and more frequent hospitalization would be more likely to be co-infected with MRSA and Pa.

Co-infection with MRSA and Pa necessitates poly-antimicrobial therapy as currently there is no available antimicrobial agent that has activity against both MRSA and Pa. Therefore, during treatment for pulmonary exacerbation, those patients could receive two concomitant potentially nephrotoxic drugs, vancomycin and tobramycin. In non-CF patients being treated in the intensive care setting, the addition of an aminoglycoside with vancomycin has been associated with increased acute kidney injury (AKI) risk [9,14]. Due to the increasing lifespan of CF patients [2] and need for greater numbers of life-long antimicrobial courses, preservation of kidney function is essential. We hypothesized that the incidence of AKI is no greater in CF patients receiving combination vancomycin and tobramycin versus either agent alone.

## Materials and methods

**Figure 1:**
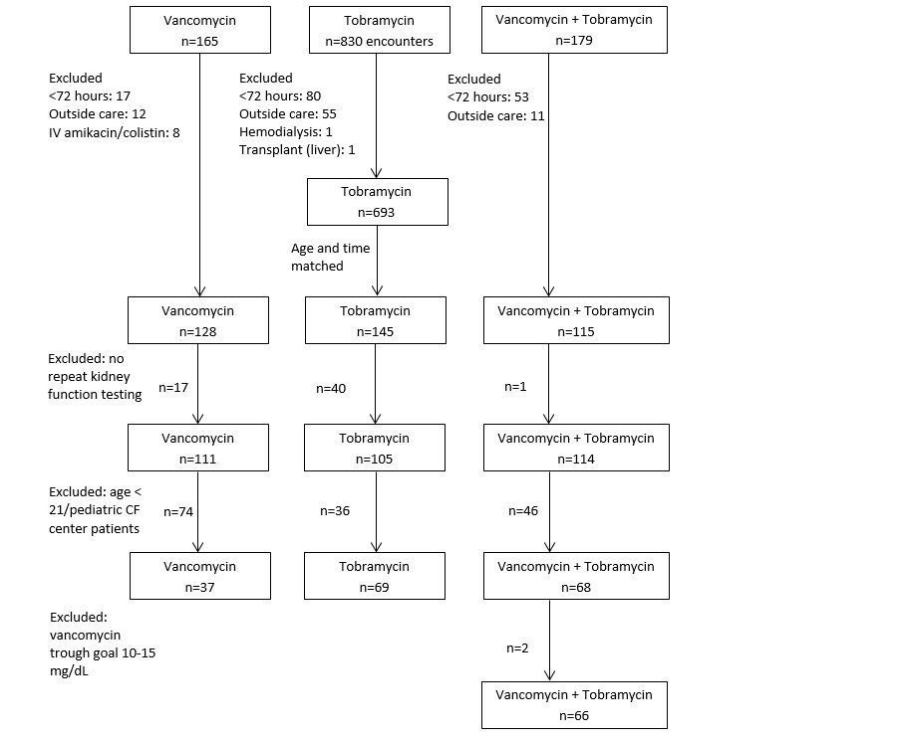
Study flow chart.

After receiving approval by the Oregon Health and Science University (OHSU) Institutional Review Board (IRB #9567), we performed a retrospective cross-sectional study of CF patients admitted to OHSU for pulmonary exacerbation over a seven-year period (1/1/2008 to 10/30/2014). Because many CF patients were admitted multiple times during the study period for treatment of separate acute pulmonary exacerbation (APE) episodes, each admission was termed an encounter. Encounters featuring therapy with intravenous tobramycin, vancomycin, or both were separated into three cohorts. Antipseudomonal antimicrobials most commonly used in combination with tobramycin at our institution are cefepime, piperacillin-tazobactam and meropenem. These agents were not included in data abstraction, thus this information is unavailable for subgroup analysis. Patients receiving inpatient care at OHSU hospital were included in data collection. Matched cohorts by age, gender and chronological time to other two groups of interest were created. There were 174 encounters from 72 unique patients included in the final analysis (Figure 1).

Patients were included in the analysis if they completed 72 hours of tobramycin and/or vancomycin, had a goal vancomycin trough of 15-20 mcg/mL, and had at least two laboratory measures of serum creatinine (SCr). Patients were excluded if they were receiving intravenous colistin or amikacin. Patients receiving care from the pediatric CF care team were excluded. Data collection included the length of stay, airway culture results, Forced Expiratory Volume at one second (FEV1) and therapeutic drug levels. A 12-hour level was collected for tobramycin, goal of 1 – 3 mg/dL. The highest levels were documented and noted if above therapeutic goal range. This unconventional random drug level monitoring practice is less specific than other methods, and may have not achieved goal values from other monitoring strategies, such as peak levels or AUC calculations. An estimation of hydration status was calculated upon admission using BUN/SCr, with a ratio of ≥20 as the cutoff for pre-renal azotemia, a presumed dehydrated state. We defined AKI a ≥1.5-fold increase in SCr per “risk” definition of RIFLE criteria (Risk, Injury, Failure, Loss of kidney function, and End-stage kidney disease). Kidney injury is defined by SCr increase 2-fold and failure by SCr increase 3-fold [15]. Urine output was not utilized as a measure of renal function as it is not routinely documented during hospitalization for APE at our institution. The analysis plan included descriptive statistics and one-way ANOVA. AKI outcome was analyzed by Chi-square or Fisher’s Exact test.

## Results

The study demographics are summarized in Table 1. Between treatment cohorts, there were no significant differences in the ages of the patients on combination therapy, nor between mean FEV1 baseline. When FEV1 baseline was stratified according to severity, there was a statistically significant difference between vancomycin, tobramycin and combination cohorts (p = 0.0005).

**Table 1:** Patient demographics by treatment group. ^§ ^p-value from ANOVA test for continuous variables and chi-square test for categorical variables.

	Vancomycin (n = 37)	Tobramycin (n = 69)	Vancomycin + Tobramycin (n = 66)	P^§^
Age, Mean (SD)	28.59 (9.5)	27.38 (8.83)	28.62 (8.9)	0.68
FEV1% baseline, Mean (SD)	57.86 (22.74)	49.72 (22.58)	51.29 (13.97)	0.12
FEV1% baseline, N (%)				0.0005
≤40	8 (21.62)	29 (42.65)	13 (20)	
40-70	19 (51.35)	29 (42.65)	48 (73.85)	
>70	10 (27.03)	10 (14.71)	4 (6.15)	

AKI outcome rates are shown in Table 2. There was no statistically significant difference in AKI occurrence overall between the three cohorts (p = 0.16). No patients had kidney failure requiring dialysis.

**Table 2:** AKI outcome rate. AKI: Acute kidney injury ^§^p-value from chi-square test or fisher’s exact test. ^Dehydration defined as BUN/SCr ≥ 20 at admission. AKI definition: ≥1.5-fold increase SCr (RIFLE) AKI injury: ≥2-fold increase SCr (RIFLE) AKI failure: ≥3-fold increase SCr (RIFLE)

	Vancomycin (n = 37)	Tobramycin (n = 69)	Vancomycin + Tobramycin (n = 66)	p^§^
AKI, N (%) total	7 (18.92)	6 (8.7)	13 (19.7)	0.16
AKI Injury, N (%)	4 (10.81)	1 (1.45)	1 (1.52)	
AKI Failure, N (%)	1 (2.7)	0 (0)	1 (1.52)	
BUN/SCr ≥ 20, N (%)	4 (10.81)	15 (21.74)	13 (19.7)	0.37
AKI, N (%) dehydrated^^^	2 (50)	1 (6.67)	1 (7.69)	0.12

For patients on vancomycin, three of the 20 total AKI events were classified in the “injury” range per RIFLE definitions and two in the “failure” range. All five injury or failure events included patients receiving vancomycin (four in the vancomycin monotherapy cohort, one in the combination cohort). All five patient encounters had vancomycin troughs that exceeded the upper limit of goal range of 20 mg/dL (22.6 – 35.3 mg/dL).

One patient treated with vancomycin met AKI criteria for five separate encounters, with a range of 1.54- to 2.7-fold increase in SCr, with the most recent incident of 2.7-fold increase denoting kidney injury and the last time this patient received vancomycin to date. In the tobramycin cohort there were six AKI events, five events qualified for risk, one injury and no failure.

## Discussion

The primary finding of this study is that no significant difference in AKI risk was seen when tobramycin and vancomycin were used in combination, compared to either agent alone in seven years of use at our institution. This supported our hypothesis that was developed based on pharmacist-managed aminoglycoside and vancomycin dosing and monitoring protocols which include monitoring renal function every 48-72 hours, however, previous studies have shown mixed results regarding increased risk of AKI when vancomycin and aminoglycosides are used. A study in 159 adults receiving vancomycin in the intensive care setting (ICU) noted an 18.89 increased odds of developing AKI when aminoglycosides were used concurrently (p = 0.002) [9]. The addition of aminoglycoside therapy also led to an increased risk of AKI in a study of 188 ICU patients receiving vancomycin for the treatment of pneumonia (OR 2.67 [1.09-6.54]; p = 0.03) [14]. The aminoglycosides that were given were not specified in either of these studies, although gentamicin use is more typical in the ICU setting, outside of treatment for patients with CF. Of note, gentamicin is more nephrotoxic than tobramycin, one double-blind randomized control study found gentamicin to be twice as likely to cause AKI compared with tobramycin [16]. A study in pediatric patients with CF looked at risk factors for developing AKI during aminoglycoside treatment [17]. This study included 82 patients with AKI, each matched with two control (non-AKI) patients. Nearly all patients received tobramycin. Vancomycin was not identified as an independent risk factor in AKI risk in CF patients on aminoglycoside therapy.

Although our results were not statistically significant, there appears to be a trend toward increased risk for patients on vancomycin, whether given with tobramycin or alone. Historically, vancomycin-associated nephrotoxicity was related to purity issues [18]. Regardless of improved manufacturing and purification processes, AKI incidences of 5% to greater than 40% have been reported in patients treated with vancomycin [8,18]. Studies have associated greater severity of illness (e.g., ICU setting), concomitant nephrotoxic agents, higher trough concentrations and longer therapy duration with increased risk for nephrotoxicity [6-9,14]. Because cystic fibrosis treatment regimens typically require both higher troughs and treatment durations greater than 10 days, lab values are monitored closely. Of note, recent reports of increased AKI risk with concomitant vancomycin and piperacillin-tazobactam suggest the need for vigilance [19,20]. Unfortunately, our study did not control the use of piperacillin-tazobactam, a commonly used anti-pseudomonal beta-lactam antimicrobial in the CF population.

Generally AKI caused by vancomycin is reversible and mild; dialysis is only required in about 3% of cases [18]. AKI from tobramycin is also typically reversible, however over-time, repeated exposures to aminoglycosides lead to reduced renal function [21]. The question of whether vancomycin and tobramycin synergistically affect renal function has been studied in vitro. Vancomycin and tobramycin both accumulate in lysosomes within proximal tubular cells of the kidney [22]. Although bound to the same cellular organelle, neither vancomycin nor tobramycin has been shown to cause a disruption in the pharmacokinetics of the other antimicrobial when both medications are used together. Evidence suggests their subcellular distributions are different and thus they are metabolized differently by the cell [22,23].

Studies comparing vancomycin and linezolid for the treatment of MRSA suspected nosocomial pneumonia have mixed results [24,25]. The ZEPHyR study compared the efficacy of linezolid and vancomycin in patients with culture positive MRSA nosocomial pneumonia [24]. This study found superior treatment outcomes with patients treated with linezolid, with cure rates of 57.6% versus 46.6% (p = 0.042) in patients treated with vancomycin. Incidence of nephrotoxicity was 8.4% with linezolid versus 18.2% with vancomycin. There was no statistical difference in mortality at 60 days. A 2015 meta-analysis compared efficacy and safety outcomes in studies comparing linezolid and vancomycin for MRSA nosocomial pneumonia [25]. For clinical cure outcomes and eradication rate, linezolid was not superior to vancomycin (RR = 1.16, 0.95-1.43) and (RR = 1.12, 0.96-1.3). Consistent with the previous study, nephrotoxicity was more frequent with vancomycin (RR = 0.5, 0.31-0.81). Recent 2016 IDSA guidelines for the treatment of hospital-acquired pneumonia recommend the use of either vancomycin or linezolid when MRSA is known or suspected [26]. These guidelines also recommend the use of hospital antibiograms for antimicrobial selection.

The MRSA treatment survey by Zobell, et al. assessed whether CF centers altered anti-MRSA antimicrobials when also treating Pa. Prescribing practices did not change in half of the respondents; however other centers report more diligence with laboratory monitoring, and some choose an alternative MRSA agent to vancomycin [12]. Standard of care at our institution continues to include vancomycin as first line therapy for MRSA in most CF patients. In our care region, vancomycin-resistant Staphylococcus aureus has not been an issue. Linezolid is the second first line agent and resistance has been observed in our CF population in patients with a history of numerous courses of linezolid. Indications for linezolid instead of vancomycin may include history of AKI, lack of therapeutic benefit from vancomycin or planned discharge on home IV therapy and concern for therapeutic drug level follow-up. Providers at OHSU continue to utilize vancomycin with concomitant tobramycin if deemed an appropriate agent.

Alternative medications for the treatment of MRSA during APE include ceftaroline, clindamycin, tigecycline, doxycycline, minocycline, and sulfamethoxazole/trimethoprim (SMX/TMP). Ceftaroline is used as an alternative therapy in patients with an unsatisfactory clinical response to vancomycin or linezolid and when vancomycin and linezolid are not options due to adverse effects or allergy. Cost-effective alternatives (Table 3) include SMX/TMP, which is used extensively for outpatient treatment of pulmonary exacerbations in CF patients [1] as it has activity against several common bacteria found in CF sputum, including MRSA, *Haemophilus influenza*, MSSA, *Stenotrophomonas maltophilia*, *Achromobacter*, and *Burkholderia spp*. Use of SMX/TMP is a risk factor for small-colony variants (SCV) of *Staphylococcus aureus* [27] thus judicious use should be considered. *S. aureus* SCVs are a slow-growing variant of MSSA, exhibit more resistance to antimicrobials and have been associated with a greater decrease in lung function in patients with CF [27]. Doxycycline is also used regularly for MRSA, MSSA and *S. maltophilia* in the outpatient setting. Unfortunately, clindamycin has a high rate of resistance which limits its usefulness, as well as risk for *Clostridium difficile* associated disease [28]. Our institution reported 2014 MRSA resistance rates of 35% to clindamycin in all patients, non-CF patients included.

**Table 3:** Antimicrobial costs. ^*^Based on most common daily adult dose. Cost codes: <$: 20 $: 20-39 $$: 40-59 $: >100

Antibiotic	Cost per day of IV therapy^*^
Amikacin	$
Tobramycin	$
Ciprofloxacin	<$
Clindamycin	<$
Linezolid	$
Trimethoprim/Sulfamethoxazole	$$
Vancomycin	$

A secondary finding of this study was that 18.6% of patients presented with an elevated BUN/SCr ratio of >20. CF patients are more prone to salt-wasting and dehydration and have higher rates of kidney stones and AKI [29]. CF patients presenting with APE frequently have elevated respiratory rates and decreased oral intake, further challenging their hydration status. This suggests intravenous fluid repletion therapy upon admission for kidney protection may be reasonable in CF patients prior to potential nephrotoxic drug therapy. BUN/SCr is an indirect measure of hydration status that can be unreliable in the CF population in general due to reduced muscle mass.

It is important to note that 8.7% of patients receiving tobramycin alone met AKI criteria. Although the practice of extended interval dosing of tobramycin reduced nephrotoxicity in pediatric patients with CF, AKI continues to be a risk when using aminoglycosides [30]. Our institution utilizes extended interval doses of 10 mg/kg every 24 hours for both adult and pediatric patients. If patients develop AKI during an aminoglycoside course, alternative agents considered for double coverage of *Pseudomonas* during APE are ciprofloxacin or inhaled tobramycin.

There are limitations to this study. First, it was a retrospective cross-sectional study and we were underpowered to make a significant conclusion about increased risk of AKI with vancomycin use. Second, typical of CF disease management, several patients were hospitalized multiple times for the same therapy. We did not control for repeat administrations of any agent as a modifier of AKI risk. Finally, antimicrobials other than vancomycin and tobramycin were not noted in the data collection, which was an oversight as concomitant medications such as piperacillin-tazobactam may have influence on AKI rates.

Although AKI rates were not statistically significantly different, the vancomycin cohorts had a higher incidence of AKI at ~19% compared with tobramycin cohort incidence of 8%. Interestingly, vancomycin and combination cohorts had similar incidence of AKI, suggesting a potential trend that vancomycin may be associated with AKI. A larger study is needed to find out definitively. Adequate hydration and close monitoring would be necessary in these patients with higher vancomycin trough goals, longer duration of therapies and concomitant therapy with piperacillin-tazobactam.

## Conclusions

Our cross-sectional study suggests that no significant interaction between concomitant vancomycin and tobramycin administration on AKI rate in CF patients hospitalized for a pulmonary exacerbation. A larger, multi-center prospective trial including other potential nephrotoxic agents, would strengthen our conclusion.
